# Poor fracture alignment equals poor outcome? Analysis of conservatively managed distal radius fractures

**DOI:** 10.1007/s00402-025-06047-9

**Published:** 2025-09-10

**Authors:** Julian Diepold, Sebastian Filipp, Florian Dussing, Gernot Steiner, Christian Deininger, Tobias Gotterbarm, Florian Wichlas

**Affiliations:** 1 Orthopaedics and Traumatology, Kepler Universitätsklinikum, Linz, Austria; 2 Johannes Kepler University of Linz, Linz, Austria; 3 Orthopaedics and traumatology, Salzburger Landeskliniken, Salzburg, Austria; 4 Department of Orthopaedics and Orthopaedic Surgery, Krankenhaus Oberndorf, Oberndorf bei Salzburg, Austria; 5 Center for Musculoskeletal Surgery, Charité - University Medicine Berlin, Berlin, Germany

**Keywords:** Distal radius fracture, Non-operative management, Fracture alignment, Range of motion

## Abstract

**Purpose:**

The NOM (non-operative management) of distal radius fractures (DRF) is influenced by various factors. This study seeks to determine whether poor fracture alignment correlates with poor outcome.

**Methods:**

Over a period of three years, a study was conducted on conservatively treated DRF involving 127 patients, 104 women (81.9%) and 23 men (18.1%). The average age was 70.6 years (SD ± 19.1; range 21 to 102 years). The patient population is categorized into two groups according to radiological healing outcomes: Group I and Group II. The classification threshold was established as (1) > 10° dorsal/volar tilt of the lateral articular surface angle. (2) Radial tilt of the anteroposterior joint surface angle exceeds 10 degrees. (3) The loss in radial height surpasses 4 mm. Patients were categorized into group II if they met two or more criteria for DRFs, while those with one or fewer criteria were placed in group I.

**Results:**

Group I exhibited superior mobility across all planes, except in radial abduction. There was also a significant improvement in the clinical scores (QuickDASH, PRWE). Patients over 70 years with anatomically healed distal radius fractures (Group I) had superior range of motion in all planes, with the exception of radial abduction. Group II exhibited significantly higher scores (QuickDASH, PRWE).

**Conclusion:**

Thus, the ultimate goal—both in younger and older patients—should remain to achieve the best possible anatomical reduction. And especially in geriatric people, anatomical repositioning demonstrates enhanced ROM and significantly improvement in patient’s satisfaction and daily functioning.

## Introduction

As patients age and comorbidities increase, it is frequently impossible to conduct the required surgery. NOM (non-operative management) is gaining significance, particularly in distal radius fractures. However, conservative treatment frequently results in subsequent tilting and malunion of the fracture. This raises the question of whether a poor fracture alignment equals poor outcome?

DRFs are one of the most common skeletal injuries in humans, but there is still no standard therapeutic regime for treating these fractures [1–3]. Even the American Academy of Orthopedic Surgeons is currently unable to provide a therapy recommendation, whether for NOM or surgical options [4]. The treatment of DRFs depends on various factors. The patient’s age, fracture morphology, surrounding soft tissues, patient demands, pre-existing medical conditions, and other factors all play a role in managing fractures [5, 6]. Both therapeutic approaches, NOM and surgical, have advantages and disadvantages [7, 8]. Major disadvantages of NOM are the cast immobilization itself and secondary dislocation. Therefore, the question is, is this relevant to the functional outcome? The likelihood of suffering a distal radius fracture is highest in boys, young men, and elderly women [9–11]. Due to modern medicine, the average age of the world’s population is constantly increasing [1]. As age advances, the prevalence of comorbidities escalates, frequently complicating surgical interventions [12]. Consequently, when patients decline or are unable to tolerate a surgical intervention due to their medical state, cast application and immobilization become the sole alternative [13]. Consequently, the NOM of DRFs remains significant [14]. For instance, complicated fractures, which necessitate surgical intervention by definition, must ultimately be managed conservatively. Consequently, follow-ups frequently lead to additional tilting and improper fracture healing.

In the current literature there is much information on comparisons between surgical versus conservative treatment of distal radius fractures, but only little information on the extent to which incorrectly healed radius fractures affect the range of motion and whether any limitation of motion is noticeable in daily life [15, 16].

This raises the question of whether displaced DRFs result in a lower ROM compared to anatomically healed fractures and whether patients with displaced fractures experience more difficulties in daily activities than those with anatomically healed fractures. The aim of this study is to investigate whether the radiological outcomes of DRFs correlate with functional outcomes and patient satisfaction.

## Materials and methods

### Patients

One hundred and twenty-seven patients with healed DRF after NOM treatment were enrolled retrospectively in two level-I-trauma centers (University Hospital Salzburg, Austria, and University Hospital Linz, Austria). The mean patient age was 70.6 years (SD ± 19.1; range 21 to 102 years); 56 times the DRF was on the right (44.1%), 64 times on the left (50.4%), and seven (5.5%) times on both sides.

In 108 patients (85.0%), the right side was dominant; in 15 patients (11.8%), the left side was dominant; in three patients (2.4%), both sides were dominant; and in one patient (0.8%), there was no documentation available. (Table 3.2.1) Local ethics committee board approval was obtained in both hospitals (Salzburg: Number: 415-E/2365/9-2018, Linz: Number: 1117/2023).

Inclusion criteria:

- DRF treated with NOM.
- Follow up > one year after osseous healing.
- Age > 18 years.

Exclusion criteria:

- DRF treated operatively.
- Time of Trauma < one year.
- Pregnancy during treatment.
- Age < 18 years.

After identifying the inclusion criteria, all additional data were taken from the digital patient archive (ORBIS (Salzburg) and KIS (Linz)). Eligible patients were then contacted by phone to participate in the study (Table 1).

**Table 1:** Characteristics of groups I and II

	Group I (*n* 67)	Group II (*n* 60)	Difference
Age ± SD	65.6 ± 19.4	77.1 ± 16.9	−11.4
Women	54	50	4
Men	13	10	3
Fx right	34	29	5
Fx left	35	36	−1
Fx both sides	2	5	−3
Right-handed	59	52	7
Left-handed	9	9	0
Both sides	1	2	−1
AO class. A	57	34	23
AO class. B	4	2	2
AO class. C	6	24	−18

### Initial reduction and treatment protocol

The general treatment principle for DRF at both clinics is as follows:

The fracture was identified using an x-ray in two planes. Before reduction, fracture gap anesthesia was performed. To reduce the DRF, the patient lies supine with 90° shoulder abduction and 90° elbow flexion. Chinese finger traps were attached to the patient’s fingers, and the upper arm was suspended from an extension frame. Weights (two- three kg) are then used to apply a longitudinal pull. This enables the physician to realign the fracture. After reduction, a cast is applied. Depending on the reduction result, the fracture morphology, the patient’s requirements, and the possibility of surgery, a decision was made to be either NOM or surgical. With NOM, the patients were examined radiologically before and after reduction and on days zero, five, 14, 28, and 42. Physiotherapy was started on the first day for fingers, elbow, and shoulder and intensified after cast removal, including the wrist. NOM-treated DRF with osseous healing of more than one year were enrolled in the study.

### Statistical analyses

All continuous variables were tested for normal distribution using the Kolmogorov-Smirnov test. If a normal distribution could be assumed, group comparisons were performed using the unpaired Student’s t-test with Welch correction in case of variance heterogeneity. For non-normally distributed variables, the Mann–Whitney U test was applied. Categorical variables were analysed using the chi-square test or Fisher’s exact test, depending on the expected cell frequencies. Correlations between radiological parameters and clinical scores were examined using the Pearson product–moment correlation coefficient for normally distributed variables and Spearman’s rank correlation coefficient for non-normally distributed variables. Statistical significance was set at *p* < 0.05, and all analyses were conducted using GraphPad Prism version X (GraphPad Software, San Diego, USA).

### Follow up protocol

Two groups were investigated and compared: anatomically healed (Group I) and malunited (Group II) DRFs. The cut-off between the groups was defined radiologically (wrist x-ray anteroposterior (AP) and lateral) at least one year after bone consolidation. The cut-off values have been selected in accordance with the instability criteria specified in the latest AWMF guidelines [17].

1. > 20° dorsal/palmar tilt of the lateral articular surface angle. Assuming that a palmar inclination of 10° is anatomical, any inclination greater than 20° towards the volar side and any inclination greater than 20° towards the dorsal side are considered non-anatomical.
2. > 10° radial tilt of the anteroposterior joint surface angle. Based on the assumption that 25 degrees of radial inclination can be assumed to be anatomical, everything > 35° and < 15° was considered pathological.
3. The radial height is reduced by more than 4 mm. We consider anything exceeding four mm to be non-anatomical.

**Fig. 1:**
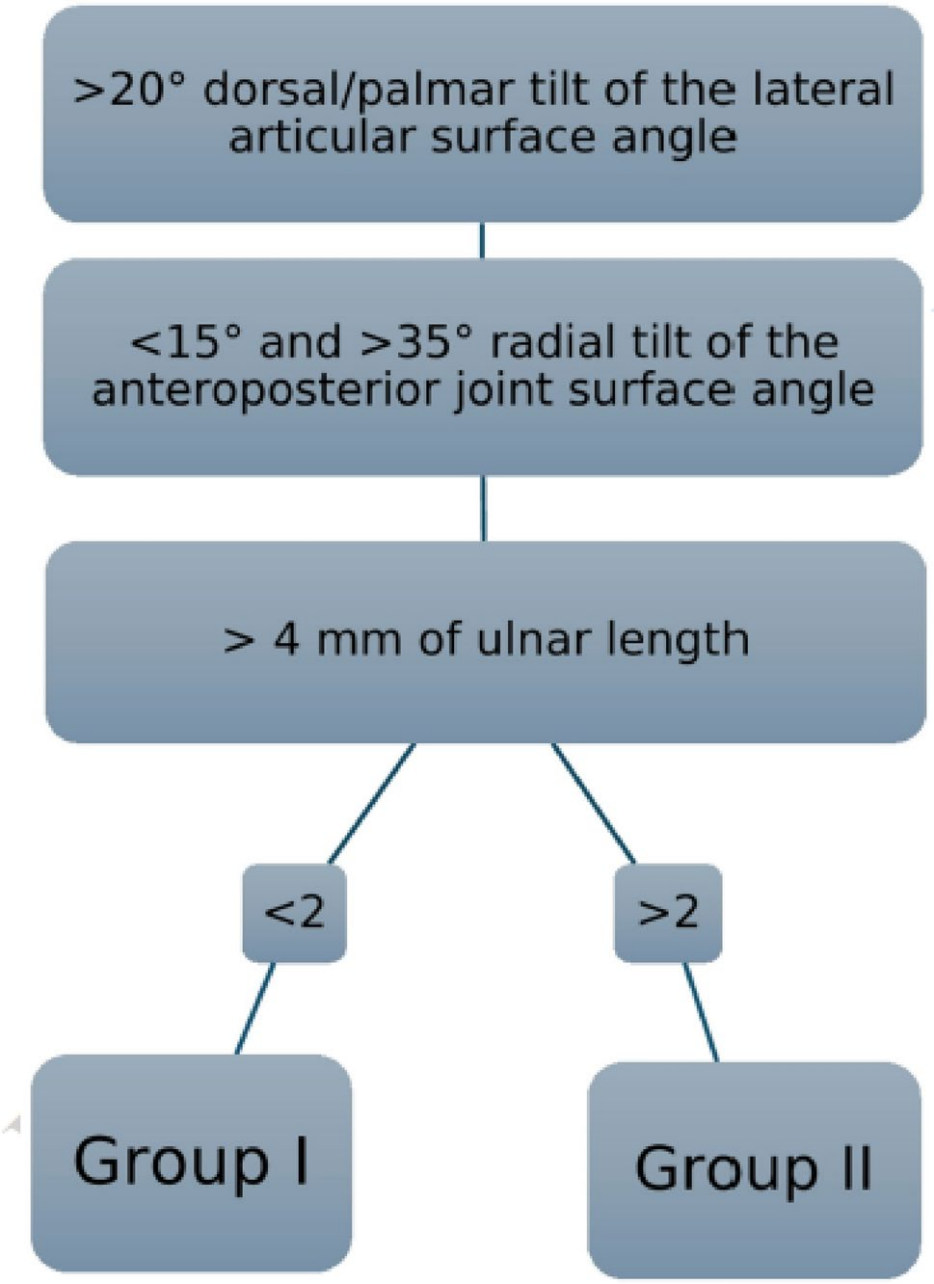
Group assignment based on radiological measurements

DRF with two or more criteria were assigned to Group II; patients with one or fewer criteria were assigned to Group I (Fig. 1)

The follow-up examination took place at least one year after osseous healing.

At follow-up, the examination included:

1. Clinical scores.
  - Short version of Disability of Arm, Shoulder, and Hand (QuickDASH) score.
  - Patient-Rated Wrist Evaluation (PRWE) Score.
  Higher scores indicate more pain and functional disability. 0 (no disability) to 100 (most severe disability/pain).
2. Clinical examination of both wrists.
  - Evaluation of the range of motion using a goniometer (flexion/extension, radial/ulnar abduction, pronation/supination).
3. Evaluate the grip strength of both sides using a dynamometer hydraulic hand dynamometer (BASELINE^®^, Fabrication Enterprises, Inc., 3 Westchester Plaza STE 111, Elmsford, NY 10523, U.S.A.); mean value of 3 compressions.
4. Radiological examination of the affected wrist, antero-posterior (AP) and lateral x-rays.

### Procedure for measuring the x-ray

Measurement of dorsal/palmar angulation in lateral x-rays:

The angle of the radius shaft axis and the line connecting the dorsal and palmar edges of the radius constitute the joint angle. Subtract ten degrees of anatomical tilt toward palmar/dorsal.

Measurement of radial inclination:

Measure the angle between the radius shaft axis and the line separating the ulnar/radial border of the radius in the A.P. x-ray.

Measurement of relative ulnar lengthening:

The ulnar end of the radius in an A.P. X-ray is aligned at a 90° angle with the radius shaft axis. Length to ulnar end measured at 90 degrees to it.

**Fig. 2:**
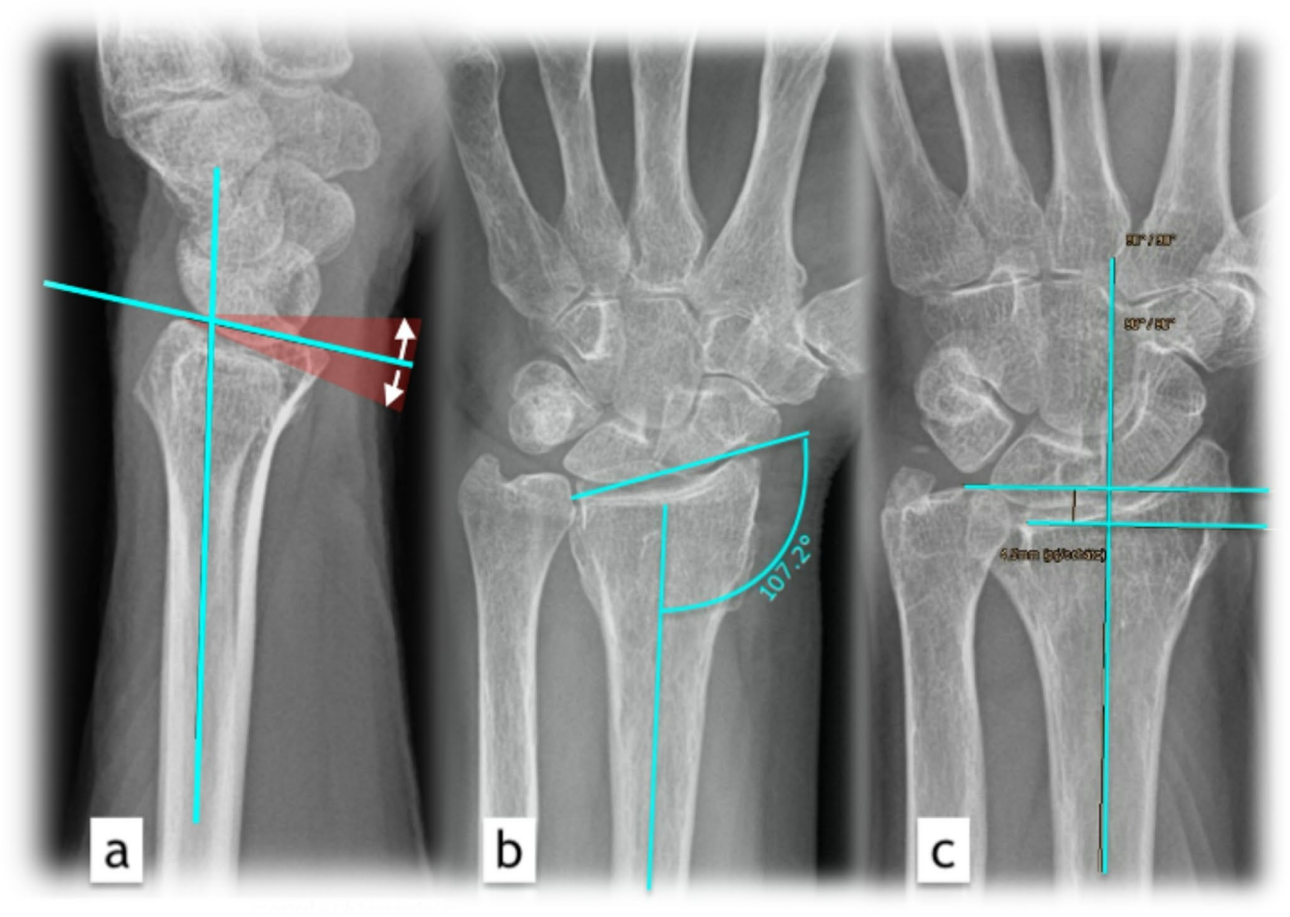
x- ray of the wrist; (**a**) palmar/dorsal inclination of the radial joint surface relative to the shaft axis, (**b**) radial inclination, angle between the distal radial joint surface relative to the shaft axis, (**c**) relative ulnar length: measurement (in mm) of the length discrepancy from the distal ulnar end to the proximal radial joint surface

The evaluated scores and radiographic and functional outcomes were compared between the two groups. (Fig. 2)

## Results

Anatomically healed DRF (Group I) had a better range of motion in all planes except for radial abduction and a significant improvement in the clinical scores (QuickDASH, PRWE). The grip strength was higher in Group I; Flexion, pronation, and ulnar abduction were significantly higher in Group I than in Group II. Mobility in the other planes (extension and supination) was higher in Group I, but not significantly. Radial abduction was higher in Group II. The different ROMs are shown in Table 2. The QuickDASH score and the PRWE score showed a significant difference. Group I was rated as “good,” and Group II was rated as “satisfactory.”

Group I showed a 15.1° extended ROM in extension/flexion, 8.4° extended ROM in pronation/supination, and 5.6° extended ROM in ulnar/radial abduction.

The full ROM in extension/flexion in Group I was 125.6°, pronation/supination 164.3°, and ulnar/radial abduction 62.1°. In Group II, the full ROM in extension/flexion was 110.5°, pronation/supination 155.8°, and ulnar/radial abduction 56.6° (Table 3).

There was no significant difference in terms of grip strength. Group I was able to apply 3.1 kg more grip strength on the fractured side than Group II. Clinical tests are shown in Table 4.

With reference to the measurements based on the x-ray, the following mean values were obtained. The dorsal tilt in group I was 9.2° less than in Group II. The palmar tilt was 1.5° less, and the radial inclination was 7.1° more in Group I. In Group II, the relative ulnar length was on average 2.4 mm longer than in Group I. There was a significant difference in the dorsal tilt, radial inclination, and total relative ulnar lengthening. Radiographic measurements are shown in Table 3. Patients in group II (*n* = 60) were on average 11.4 years older than in group I (*n* = 67).

The subgroup analysis revealed that patients over the age of 70 with anatomically healed DRF (Group I) exhibited improved range of motion in all planes, with the exception of radial abduction. Significant improvements were observed in palmar flexion, ulnar abduction, QuickDASH and PRWE scores. The grip strength was greater in Group I but showed no significant difference. ROM and clinical tests are shown in Table 5.

The subgroup indicated that patients aged under 70 had a better range of motion in palmar flexion, dorsal extension, ulnar abduction and supination. No significant difference has been observed in all ROMs and clinical scores. ROM and clinical tests are shown in Table 6.

A comparison of the data from the older and younger patients revealed a significant difference in extension, pronation, supination, and radial and ulnar abduction, in the grip strength and QuickDASH scores. However, it was observed that only pronation and the PRWE score demonstrated a lack of statistical significance.

**Table 2:** Range of motion of the fractured wrist

	Group I (*N* 67)	Group II (*N* 60)	Difference	*P* -Value
Flexion° ± SD	62.0°± 11.1	53.0°± 16.8	9.0	<0.05
Extension° ± SD	63.6°± 13.8	57.4°± 16.0	6.2	0.09
Pronation° ± SD	87.0°± 8.4	82.5°± 15.0	4.5	<0.05
Supination° ± SD	77.2°± 18.4	73.3°± 22.1	3.9	0.48
Radialabduction° ± SD	22.7°± 7.6	25.1°± 11.3	−2.4	0.43
Ulnarabduction° ± SD	39.2°± 11.9	31.5°± 12.5	7.7	<0.05

* < 0.05 significant

**Table 3:** Radiographic measurements of the fractured wrist

	Group I (*n* 67)	Group II (*n* 60)	Difference	*P*-value
Dorsal tilt (°) ± SD	8.11° ± 6.41	17.30° ± 7.81	−9.2	<0.05
Palmar tilt (°) ± SD	10.23° ± 5.43	11.76° ± 7.81	−1.5	0.91
Radial inclination (°) ± SD	23.58° ± 5.24	16.44° ± 8.48	7.1	< 0.05
Total relative ulnar lengthening (mm) ± SD	1.47 mm ± 1.47	3.89 mm ± 3.19	−2.4 mm	< 0.05

* < 0.05 significant

**Table 4:** Clinical tests and grip strength of the fractured wrist

	Group I (*n* 67)	Group II (*n* 60)	Difference	*P*-value
Quick-dash ± SD	10.8 ± 15.1	19.6 ± 20.4	8.8	< 0.05
Prwe ± SD	9.9 ± 14.6	17.4 ± 20.1	7.5	< 0.05
Grip strength ± SD	25.0 ± 12.9	21.9 ± 15.5	3.1	0.06

* < 0.05 significant

**Table 5:** Range of motion and clinical tests in subgroups over 70 years

	Subgroup I > 70 YEARS	Subgroup II > 70 YEARS	Difference	*P* -Value
Flexion° ± SD	59.8°± 11.5	49.9°± 16.6	9.9	<0.05
Extension° ± SD	58.2°± 12.2	54.6°± 16.0	3.6	0.54
Pronation° ± SD	85.9°± 10.5	81.3°± 16.7	4.6	0.11
Supination° ± SD	73.3°± 21.2	70.5°± 23.3	2.9	0.76
Radialabduction° ± SD	20.6°± 7.5	23.1°± 10.8	−2.5	0.54
Ulnarabduction° ± SD	36.0°± 12.6	28.9°± 11.2	7	<0.05
Quick-dash ± SD	12.1 ± 17.2	22.7 ± 20.8	−10.6	<0.05
Prwe ± SD	9.6 ± 15.9	20.1 ± 21.4	−10.4	<0.05
Grip strength ± SD	20.1 ± 9.3	17.2 ± 9.4	2.9	0.09

* < 0.05 significant

**Table 6:** Range of motion and clinical tests in subgroups under 70 years

	Subgroup I < 70 YEARS	Subgroup II < 70 YEARS	Difference	*P* -Value
Flexion° ± SD	64.4°± 10.2	61.6°± 14.7	2.8	0.72
Extension° ± SD	69.4°± 13.3	65.1°± 13.4	4.3	0.48
Pronation° ± SD	88.3°± 5.2	85.9°± 8.4	2.3	0.31
Supination° ± SD	81.5°± 13.9	81.3°± 16.3	0.3	0.87
Radialabduction° ± SD	25.6°± 8.4	30.8°± 11.1	−5.2	0.06
Ulnarabduction° ± SD	42.7°± 10.1	38.4°± 13.6	4.3	0.02
Quick-dash ± SD	9.4 ± 12.5	11.1 ± 16.8	−1.7	0.93
Prwe ± SD	10.2 ± 13.4	10.1 ± 14.2	0.1	0.76
Grip strength ± SD	30.3 ± 14.3	35.2 ± 21.0	−4.9	0.50

## Discussion

Anatomically healed DRFs showed better results in terms of ROM and clinical scores. In terms of patient demographics, there are young, healthy patients who put high demands on their affected wrists, as well as elderly patients who require care and seriously ill patients with very limited demands [2, 18, 19]. The patients’ age does not directly reflect the requirements imposed on the injured wrist. Currently, a patient’s biological age may not align with their chronological age. Restoring rapid wrist function is particularly crucial for elderly individuals [20]. The impacted wrist must possess sufficient functionality to enable the patient to perform self-care activities. The presence or absence of mobility constraint is not very significant. Frequently, x-ray follow-ups show a significant tilting and incorrect healing of the fracture. However, the patients report only a minimal limitation in daily life. This scenario raises the question of which parameters should be used to assess the functional outcome of the fractured wrist [21].

Also, other study groups like Arora et al. showed that achieving anatomical reconstruction did not convey any improvement in terms of the range of motion or the ability to perform daily living activities [22].

Furthermore Quadlbauer et al. were unable to identify a significant difference between an unacceptable (> 2 mm) and an acceptable (< 2 mm) ulnar variance in terms of patient-reported outcome measurements, grip strength, ROM, and discomfort [20]. 

A systematic review of Diaz-Garcia et al. supports the treatment with cast immobilization. Comparing the most popular methods of treating a DRF—the volar locking plate system, joint bridging external fixation, nonbridging external fixation, percutaneous Kirschner wire fixation, and cast immobilization—the systematic review suggests that despite worse radiographic outcomes associated with conservative treatment, functional outcomes were not different from those of surgically treated groups for patients age 60 and older [23].

Also, Ju et al. concluded that surgical and nonsurgical methods produce similar results in the treatment of DRFs in patients 65 years and older [24].

Young et al. showed the NOM of distal radius fractures in patients with low functional demands yields satisfactory outcomes. Also, the functional outcome and return to the previous activity level, regardless of the radiographic result, have been observed [25].

As Leixnering et al. showed that indications of DRFs are dependent on many factors that influence the choice of surgical treatment. It has been shown that the conservative method is a reasonable alternative. Especially in elderly patients, with low demands and osteoporosis, an operation must be carefully considered [26]. 

In our study cohorts, it was shown that there is a significant difference in flexion (9°), pronation (4.5°), and ulnar abduction (7.7°). For the remaining parameters, the difference is not significant. In relation to the PRWE score, a significant difference can be seen here. Group I had a mean value of 9.9 (SD ± 14.6), and group II had a mean value of 17.4 (SD ± 20.1). The clinical score showed a significant difference between the two groups.

The QuickDASH also showed a significant difference. In Group I, the QuickDASH was 10.8 (SD ± 15.1), and in Group II, it was 19.6 (SD ± 20.4). At the detailed classification of the QuickDASH, Group I is good and Group II is satisfactory.

Thus it’s interesting to observe that Group II, with patients over 70 years, exhibits a significantly higher QuickDASH and PRWE than patients under 70 years. This demonstrates the critical nature of anatomical repositioning, particularly in geriatric patients. Referring to the limitations of the study, one of the difficulties was the cognitive state of the patients, as it was often difficult to evaluate the questionnaires, as many of the patients had dementia and were already living in a retirement home and had only limited self-care. It was also difficult to determine whether a limitation in daily life was mainly caused by the affected wrist itself or whether the multiple primary illnesses were responsible for the limitation. The radiological measurement of the malunion radius fractures also revealed the difficulty of determining the exact angles, as the incorrect healing often changed the radiocarpal joint situation so significantly that measurement was only possible to a limited extent.

Moreover, participation in the study was voluntary. Consequently, the possibility of a selection bias cannot be discounted.

Therefore, addressing NOM of distal radius fracture, the following recommendations can be made: Anatomically healed distal radius fractures have mainly a better range of motion in nearly all planes. Nonetheless, it is statistically significant only in flexion, pronation, and ulnar abduction. The enhanced mobility significantly influenced patient well-being and daily functionality especially in patients over 70 years of age. Consequently, the determination regarding subsequent treatment especially in geriatric patients must remain individualized, patient-specific, and risk-oriented.

The ultimate goal—both in younger and older patients—should remain to achieve the best possible anatomical reduction. And especially in geriatric people, anatomical repositioning demonstrates enhanced ROM and significantly improvement in patient’s satisfaction and daily functioning.

## Data Availability

No datasets were generated or analysed during the current study.
